# Large Doses Ammonia Carbonate in the Suffocative Stages of Acute Pulmonary Diseases

**Published:** 1886-08-20

**Authors:** Benj. H. Riggs

**Affiliations:** Selma, Alabama


### LARGE DOSES AMMONIA CARBONATE IN THE SUFFOCATIVE STAGES OF ACUTE PULMONARY DISEASES.

BY BENJ. H. RIGGS, M. D., SELMA, ALA.

The usefulness of carbonate of ammonia as a heart tonic is not fully appreciated. It acts like alcohol, but in a different way; both are useful stimulants to a weak heart, and are a present help in times of collapse or depression—such as the shock of gunshot wounds, or the temporary depression which accompanies acute diseases or its coexistence with an overwhelmed state of the vitality by a too great degree of morbific poison. Alcohol stimulates the heart, but it is accompanied by the paralyzing and stupefying effect ultimately produced by a large injection of alcohol, degrees, more or less pronounced, of drunkenness. This is an objection to its use in some states of the system. On the other hand, carbonate of ammonia is simply a cardiac stimulant, but, while not stupefying, it is not near so palatable as good brandy or champagne, and therefore useless in a very irritable stomach, and in large doses it purges, nauseates, and ultimately defibrinates the blood. Each one of these heart supporters fills a want, and neither one can take the place of the other. Carbonate of ammonia answers a useful purpose in the suffocative stages of bronchitis, pneumonia, cardiac asthma, etc., of the adult—if an irritable stomach does not prohibit its use. Sometimes we can utilize the aromatic spirits in these cases where we cannot give carbonate. But if you have a good stomach, you can give carbonate of ammonia in 20 grain doses, well diluted with water, in these cases, and repeat it every two hours for twelve to twenty-four hours, with good results. Ten grains repeated every two hours is a less nauseating dose. It is proper to combine this with a syrup of tolu, but not with syrup of squills, on account of the latter containing more or less acetic acid.

But it is especially in the suffocating stages of capillary bronchitis, in infants within the two years, that large doses of carbonate of ammonia are especially useful. I cannot emphasize this too much. It is the great remedy. It has magically relieved many cases in my hands, and driven away the rapid approach of death. Physicians are hardly prompt enough to observe the approach of this drowning process. Its approach is at times very insidious. If you go to an infant that is restless, maybe it has been so all night or all day; there is a distressed look on his little face, his lips are white, around his eyes a dark ring, the cheeks perhaps have a purple flush that comes and goes, he may be hot to the touch, or he may be cold and sweaty, he coughs a great deal, or he coughs very little, oftenest this, and his cough is suppressed, and seems to hurt him; given these symptoms, you can safely look out for pulmonary congestion. If you auscultate the chest, you will find coarse rales, but absent or deficient vesicular rales.

Here your great remedy is carbonate of ammonia; it will save life. It is very important that you give it in this particular way:

℞. Ammonia carbonate........................grs. 16,

Syrup tolutan, } aa..........................℥ I.

Aqua, }

M.

Sig. One teaspoonful every two hours—given in this way: Take a *silver* teaspoonful of the mixture and put it in a teacup or glass, and add 3 teaspoonfuls of cold water to it, and give 1 teaspoonful of this diluted dose every thirty minutes. This will do for a child from two months old to eight months old. After that the dose may be increased to 3 or 5 grains, thus diluted.

This half-hour dose is very important: it is all important. You thus keep up a gentle and constant support to the over-burdened heart. It is more useful than every two-hour doses.

I emphasizes silver spoon, as the alloy of other spoons contains more or less copper, which will soon form cuprum ammoniatum with the solution. We ought never to give calomel with this solution for fear of chemical change in ammoniated mercury. We can give quinine though, even if it is changed into carbonate, and ought never to fail to do it after the next night.

To make this treatment a perfect success we ought to combine with it the hot mustard foot-bath repeated every four hours, and applying mustard-mush poultices to front and rear of chest, repeating every two hours, and allowed to stay on only twenty minutes at a time, if *strong.—Alabama Medical Journal.*

